# A Mixture of *Morus alba* and *Angelica keiskei* Leaf Extracts Improves Muscle Atrophy by Activating the PI3K/Akt/mTOR Signaling Pathway and Inhibiting FoxO3a In Vitro and In Vivo

**DOI:** 10.4014/jmb.2306.06012

**Published:** 2023-08-25

**Authors:** Hyun Hwangbo, Min Yeong Kim, Seon Yeong Ji, Da Hye Kim, Beom Su Park, Seong Un Jeong, Jae Hyun Yoon, Tae Hee Kim, Gi-Young Kim, Yung Hyun Choi

**Affiliations:** 1 Anti-Aging Research Center, Dong-eui University, Busan 47340, Republic of Korea; 2 Hamsoa Pharmaceutical Co., Ltd., Iksan 54524, Republic of Korea; 3 Department of Marine Life Science, Jeju National University, Jeju 63243, Republic of Korea; 4 Department of Biochemistry, Dong-eui University College of Korean Medicine, Busan 47227, Republic of Korea

**Keywords:** Muscle atrophy, *Morus alba*, *Angelica keiskei*, FoxO3a, PI3K/Akt/mTOR

## Abstract

Muscle atrophy, which is defined as a decrease in muscle mass and strength, is caused by an imbalance between the anabolism and catabolism of muscle proteins. Thus, modulating the homeostasis between muscle protein synthesis and degradation represents an efficient treatment approach for this condition. In the present study, the protective effects against muscle atrophy of ethanol extracts of *Morus alba* L. (MA) and *Angelica keiskei* Koidz. (AK) leaves and their mixtures (MIX) were evaluated in vitro and in vivo. Our results showed that MIX increased 5-aminoimidazole-4-carboxamide ribonucleotide-induced C2C12 myotube thinning, and enhanced soleus and gastrocnemius muscle thickness compared to each extract alone in dexamethasone-induced muscle atrophy Sprague Dawley rats. In addition, although MA and AK substantially improved grip strength and histological changes for dexamethasone-induced muscle atrophy in vivo, the efficacy was superior in the MIX-treated group. Moreover, MIX further increased the expression levels of myogenic factors (*MyoD* and *myogenin*) and decreased the expression levels of E3 ubiquitin ligases (*atrogin-1* and muscle-specific RING finger protein-1) in vitro and in vivo compared to the MA- and AK-alone treatment groups. Furthermore, MIX increased the levels of phosphorylated phosphoinositide 3-kinase (PI3K), protein kinase B (Akt), and mammalian target of rapamycin (mTOR) that were reduced by dexamethasone, and downregulated the expression of forkhead box O3 (FoxO3a) induced by dexamethasone. These results suggest that MIX has a protective effect against muscle atrophy by enhancing muscle protein anabolism through the activation of the PI3K/Akt/mTOR signaling pathway and attenuating catabolism through the inhibition of FoxO3a.

## Introduction

Skeletal muscle plays an important role in physical activities, such as mobility and movement, while interacting with other tissues, such as bone, heart and fat tissue, through the secretion of myokines [1-3]. In addition, skeletal muscle mass is maintained through the balanced control of muscle protein synthesis and degradation, and their imbalance leads to increased muscle damage and atrophy [4-6]. The loss of skeletal muscle due to atrophy is closely associated with aging, physical inactivity, various chronic diseases, and drug abuse [7, 8]. Muscle atrophy has a major impact on physical activity, as it limits body movement due to muscle weakness, resulting in movement disorders [4, 9]. As the elderly population continues to grow, social interest in muscle atrophy is also increasing, and it has become necessary to find ways to prevent and treat this disorder.

Recent studies have reported that AMP-activated protein kinase (AMPK), which plays an important role in muscle metabolism and transcriptional regulation, is involved in muscle wasting [10-12]. AMPK is a regulator of protein catabolism and anabolism, and excessive AMPK activity results in muscle loss and decreased mammalian target of rapamycin (mTOR) signaling [13, 14]. In addition, the administration of a synthetic AMPK inducer promotes muscle fiber atrophy by stimulating the ubiquitin-proteasome system and autophagy [15, 16].

Glucocorticoids (GCs), such as dexamethasone, are anti-inflammatory and immunosuppressive agents used to treat diseases including rheumatoid arthritis and bronchial asthma. GCs bind to glucocorticoid receptors and promote the expression of target factors by activating GC response elements [17]. One of the target organs of GCs is skeletal muscle, and skeletal muscle atrophy is a side effect of high-dose or long-term GC administration [18,19]. Inactivation of the phosphoinositide 3-kinase (PI3K)/protein kinase B (Akt) pathway by GCs leads to the activation of forkhead box O3 (FoxO3a), a transcription factor for E3 ubiquitin ligase, resulting in muscle atrophy [20,21]. Meanwhile, activation of the E3 ubiquitin ligases *atrogin-1* and muscle-specific RING finger protein-1 (*MuRF1*) accelerates muscle protein degradation [5, 22]. The importance of E3 ubiquitin ligase has been demonstrated by examining the altered regulation of muscle atrophy in *atrogin-1*- and *MuRF1*-null mouse models [7, 23]. In addition, the levels of myogenesis-related factors, such as *MyoD*, *myogenin*, myogenic factor 5 (Myf5) and myogenic regulatory factor 4 (Mrf4), are inversely proportional to elevated GC levels [17, 24].

*Morus alba* L. (mulberry) has been used in traditional medicine to treat hyperglycemia, hypertension, dyslipidemia, and diabetes [25-27]. Many researchers have confirmed that *M. alba* contains various active ingredients, such as polyphenols, flavonoids, anthocyanins, and polysaccharides, which lower lipid levels while also regulating blood sugar levels and antioxidant and anti-inflammatory activities [27, 28]. *Angelica keiskei* Koidz. (known as 'Myeong-Il Yeob' in Korea and 'Ashitaba' in Japan) has been used to treat diseases such as asthma, chronic hepatitis, diabetes, gastritis, hypertension, and obesity. It has also been used in folk medicine to relieve muscle and joint pain [29, 30]. *A. keiskei* contains more than 100 types of active substances, including various flavonoids, and several studies have reported its anti-inflammatory, anti-obesity, antioxidant, anticancer, diabetes-modifying, and hepatoprotective activities [30, 31]. Although leaf extracts of *M. alba* and *A. keiskei* were known to exert positive effects on various physiological activities, their effects on muscle atrophy had not been investigated. In this study, we therefore sought to elucidate the mechanisms underlying the ameliorative effects of ethanol extracts of *M. alba* (MA) and *A. keiskei* leaves (AK) and their mixtures (MIX) on muscle atrophy.

## Materials and Methods

### Materials

Dulbecco’s modified Eagle’s medium (DMEM), fetal bovine serum (FBS), and penicillin-streptomycin were purchased from Welgene Inc. (Korea). Giemsa solution was purchased from Samchun Chemical Co., Ltd. (Korea), while 5-aminoimidazole-4-carboxamide ribonucleotide (AICAR), dexamethasone, and hematoxylin and eosin were purchased from Sigma-Aldrich (USA). In addition, 3-(4,5-dimethylthiazol-2-yl)-2,5-diphenyltetrazolium bromide (MTT), dimethyl sulfoxide (DMSO), TRIzol, bovine serum albumin (BSA), 3-hydroxy-3-methylbutyric acid (HMB), and xylene were obtained from Thermo Fisher Scientific (USA). SuPrimeScript RT Premix and AccuPower 2X GreenStar qPCR Master Mix were purchased from Genetbio Co., Ltd. (Korea) and Bioneer (Korea), respectively. Anti-myosin heavy chain (MyHC) antibody was purchased from R&D Systems (USA). Primary and secondary antibodies against MyoD, myogenin, atrogin-1, and FoxO3a were provided by Santa Cruz Biotechnology, Inc. (USA). Formaldehyde was purchased from Junsei Chemical Co., Ltd. (Japan). Paraffin was purchased from Leica Biosystems (Germany). The anti-MuRF1 antibody was purchased from Abcam (UK) and the anti-phosphorylated (p)-mTOR (Ser 2448), p-PI3K (Tyr 458), and p-Akt1/2/3 (Ser 473, Ser 474, Ser 472) antibodies were obtained from Cell Signaling Technology (USA). Finally, 3,3-diaminobenzidine (DAB) was purchased from Vector Laboratories Inc. (USA).

### Preparation of MA, AK, and MIX

Ethanol extracts of MA, AK, and MIX used in this experiment were provided by Hamsoapharm Co., Ltd.(Korea). MA and AK were purchased from the Sancheong-gun Sericulture Agricultural Cooperative (Korea) and The One Nature (Korea), respectively. To prepare the extracts, each sample was selected and prepared according to the following process: After adding 20 times 40% EtOH and circulating extraction at 55–60°C for 5–6 h, a filtration process was performed. Thereafter, the filtrate was concentrated under reduced pressure for 20 brix or more, sterilized at 90°C or more for 10 min or more, and then filtered (40 mesh). The filtered concentrate was subjected to final spray drying. The MIX was prepared by mixing 11% of MA and 89% of AK and proceeding in the same manner as above.

### Cell Culture and Myotube Differentiation

The mouse C2C12 myoblasts used in this study were purchased from the American Type Culture Collection (USA). The cells were cultured in DMEM with FBS and penicillin-streptomycin at 37°C with humidified 5% CO_2_. To induce myotube differentiation, the cells were seeded in 6-well plates and the medium was replaced with DMEM containing 2% horse serum until the cells reached > 90% confluence. The medium was changed every 2 days during the induction of differentiation. The morphology of the myotubes was confirmed by staining with Giemsa solution. Images of myotubes were captured using the EVOS FL Auto 2 imaging system (Thermo Fisher Scientific, USA) and the thickness of the myotubes was measured using Celleste Image Analysis software (Thermo Fisher). For each sample, at least 100 myotubes were analyzed in eight random fields.

### Cell Viability

Cells (1 × 10^5^ cells/well) were seeded in 6-well plates and incubated for 24 h. They were pretreated with MA, AK, and MIX (400 μg/ml) provided by Hamsoapharm Co., Ltd. for 1 h, and then treated with AICAR (1 mM) for 24 h. To determine cell viability using the MTT assay, an MTT solution (5 μg/ml) was added to each well and the cells were incubated for 2 h. The formazan crystals produced during the reaction were dissolved in DMSO and the absorbance was measured at 540 nm using a microplate reader (Molecular Devices, USA) [32].

### Quantitative Reverse Transcription Polymerase Chain Reaction (qRT-PCR)

C2C12 myotubes and gastrocnemius muscle tissues were chopped and washed repeatedly, and total RNA was extracted using TRIzol reagent. RNA samples quantified in equal amounts of 1 μg were reverse transcribed into complementary DNA using SuPrimeScript RT Premix. mRNA expression was measured by performing qRT-PCR using the CFX Duet Real-Time PCR system with AccuPower 2X GreenStar qPCR Master Mix and specific primers (Table 1). Data were analyzed using delta delta cycle threshold methods and normalized to GAPDH (glyceraldehyde-3-phosphate dehydrogenase) expression levels.

### Immunofluorescence

C2C12 myotubes were treated with MA, AK, MIX, or AICAR for 24 h and MyHC expression levels were measured. After incubation, the cells were fixed with ice-cold methanol and blocked with 5% BSA. The cells were then incubated with a specific primary antibody (1:100 in 2.5% BSA), followed by a secondary antibody (goat anti-rabbit IgG cross-absorbed secondary antibody conjugated to Alexa Fluor 488). Cell fluorescence was observed using an EVOS FL Auto 2 imaging system (Thermo Fisher) [33].

### Animals and Experimental Design

Six-week-old male Sprague Dawley (SD) rats were purchased from Koatech (Korea). The SD rats were housed in a temperature- and light-controlled room (23 ± 2°C with 12 h light/dark cycle). All the animals were provided with free access to food and water throughout the experiment. All animal care and experimental procedures were approved by the Institutional Animal Care and Use Committee of Dong-eui University (No. C2021-011). After a week-long acclimatization period, the SD rats were randomly divided into six groups (n = 7/group): the control group (C) was administered saline; the dexamethasone-treated group (D) was administered dexamethasone (600 μg/kg/day); the dexamethasone/MA group (MA) was administered dexamethasone (600 μg/kg/day) and MA (100 mg/kg/day); dexamethasone/AK group (AK) was administered dexamethasone (600 μg/kg/day) and AK (100 mg/kg/day); the dexamethasone/MIX group (MIX) was administered dexamethasone (600 μg/kg/day) and MIX (100 mg/kg/day); and the dexamethasone/PC group (PC) was administered dexamethasone (600 μg/kg/day) and 3-hydroxy-3-methylbutyric acid (HMB, 320 mg/kg/day). The animals were administered DEX, AK, MA, or MIX once daily for 10 days. Body weight was measured every alternate day, and the gastrocnemius and soleus muscles were harvested on the last day of the experiment, weighed, measured for thickness, and stored for analysis.

### Measurement of Grip Strength

The grip strength of all the animals was measured using a grip strength meter on the day before the end of the experiment. A grip-strength meter was used to measure the maximum strength of rats. To make the rats grab the grid, the tail of the mouse was held and positioned over it. The rats were pulled at a constant speed until they released the grid. This measurement was recorded five times per animal and expressed as the average value [34].

### Hematoxylin and Eosin (H&E) Staining

The gastrocnemius tissue was fixed in 4% formaldehyde for 24 h, dehydrated with ethanol and xylene, and embedded in paraffin. The paraffin-embedded muscle tissue blocks were cut into 4-μm-thick sections and stained with H&E. To measure the cross-sectional area (CSA), the stained muscle tissue was photographed using the EVOS FL Auto 2 imaging system (Thermo Fisher) and analyzed using the Celleste Image Analysis software in five random areas of each section [35].

### Immunohistochemistry Analysis

For immunohistochemical staining, gastrocnemius muscle sections were deparaffinized and rehydrated, and specific primary antibodies were applied. *MyoD*, Myogenin, *MuRF1*, MAFbx/*atrogin-1*, p-mTOR and FoxO3a antibodies diluted 1:200 were used for the immunoreaction and visualized by DAB staining. The stained sections were observed using an EVOS FL Auto 2 imaging system (Thermo Fisher) [36].

### Statistical Analysis

The results were expressed as the mean ± SD from triplicate experiments and analyzed with GraphPad Prism 8 (Version 8.4.2; GraphPad Inc., USA). Statistical differences were determined using one-way analysis of variance for multiple comparisons, followed by Tukey’s post-hoc test. Statistical significance was set at *p* < 0.05.

## Results

### MIX Modulates Myogenic Differentiation and Muscle Atrophy-Related Genes

**Fig. 1:**
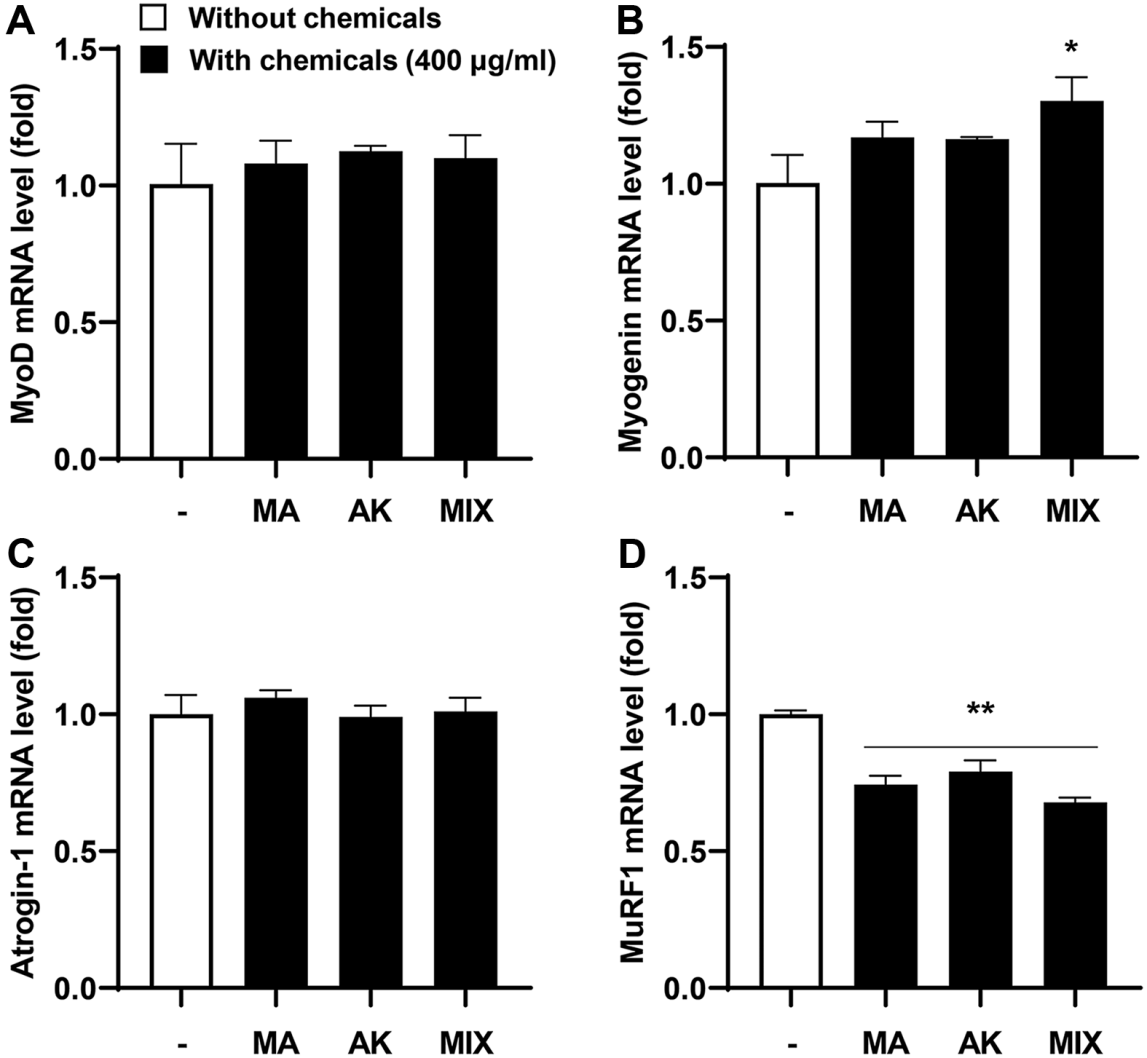
Effects of MA, AK, and MIX on the mRNA expression levels of myogenic genes and atrophy-related genes in C2C12 myotubes. Relative mRNA expression levels of *MyoD* (**A**) *myogenin* (**B**) *atrogin-1* (**C**) and *MuRF1* (**D**) were assessed using qRT-PCR. Data are expressed as means ± SD. *p < 0.05 and **p < 0.01 compared with the control.

First, we investigated the changes in mRNA expression following MA, AK, and MIX treatment of C2C12 myotubes. For this purpose, C2C12 myotubes were treated with 400 μg/ml of MA, AK, or MIX for 24 h, followed by examination of the expression of *MyoD* and *myogenin* related to myogenesis, and *atrogin-1* and *MuRF1* related to muscle atrophy. As shown in Fig. 1, MIX significantly increased the expression of *myogenin*, and MA, AK, and MIX dramatically reduced the expression of *MuRF1*. Although there were no significant changes in *MyoD* and *atrogin-1* expression, these results suggest that the MIX extract has potential protective effects against muscle atrophy.

### MIX Prevents AICAR-Induced C2C12 Myotube Atrophy

**Fig. 2:**
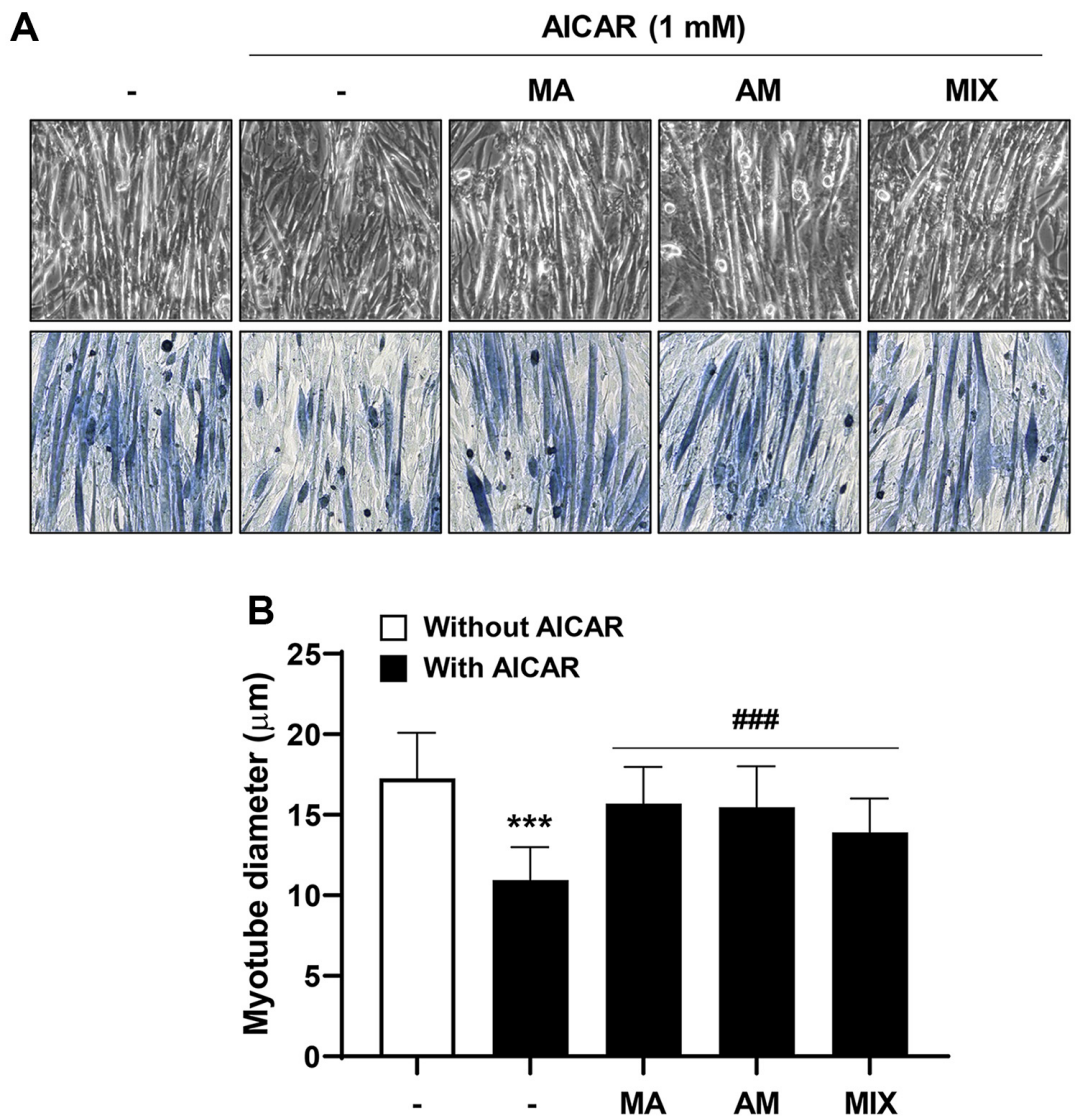
Effects of MA, AK, and MIX on the thickness of myotubes decreased by AICAR in C2C12 cells. (**A**) Morphological observation of myotubes (upper panels) and myotube images with Giemsa staining (lower panels). (**B**) The diameter of C2C12 myotubes were determined. Data are expressed as means ± SD. ***p < 0.001 compared with the control. ^###^*p* < 0.001 compared with AICAR-treated cells.

**Fig. 3:**
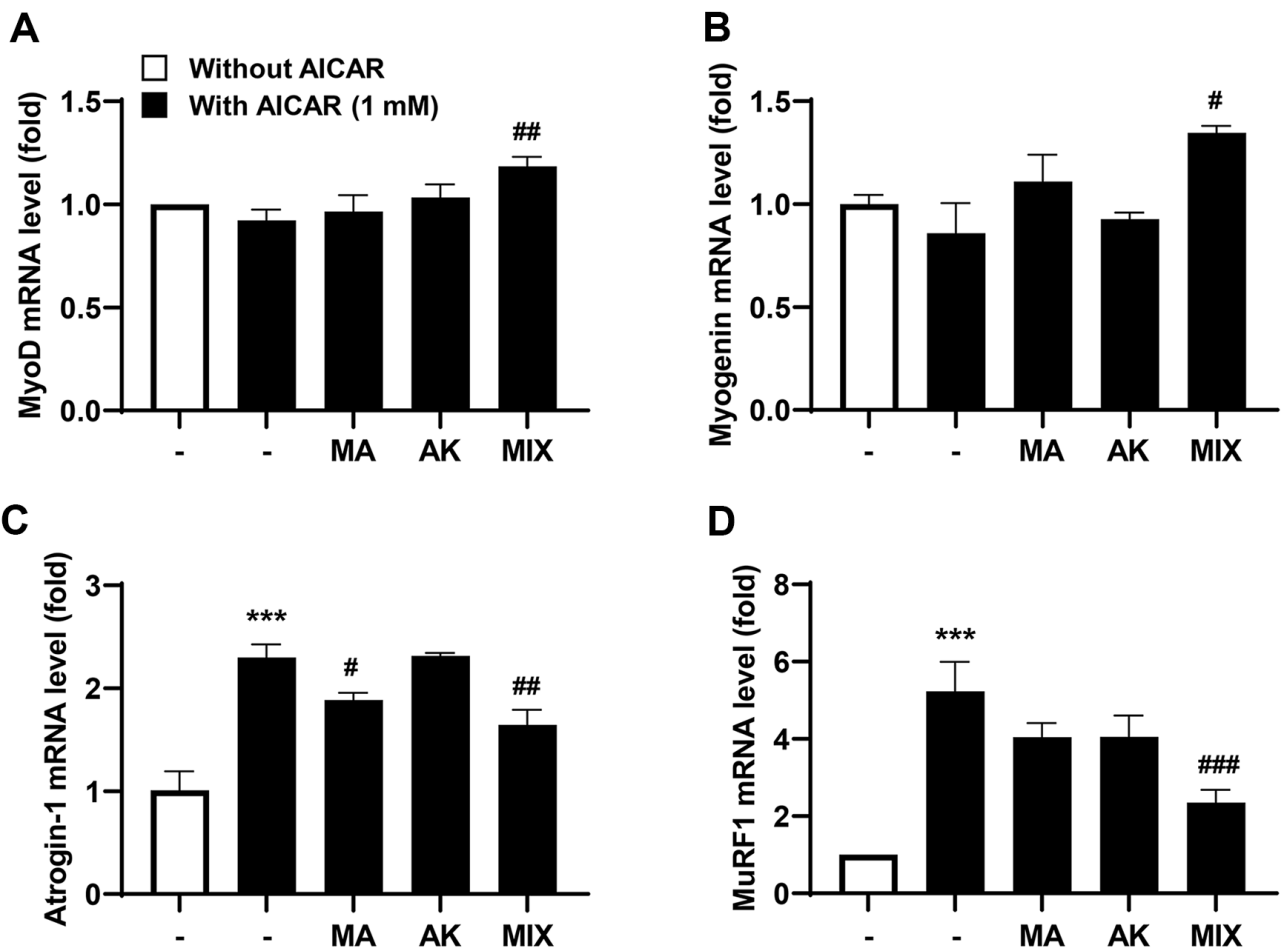
Effects of MA, AK, and MIX on the mRNA expression levels of myogenic genes and atrophy-related genes altered by AICAR in C2C12 myotubes. Relative mRNA expressions levels of *MyoD* (**A**) *myogenin* (**B**) *atrogin-1* (**C**) and *MuRF1* (**D**) were assessed by qRT-PCR. Data are expressed as means ± SD. ***p < 0.001 compared with the control. ^#^*p* < 0.05, ^##^*p* < 0.01, and ^###^*p* < 0.001 compared with AICAR-treated cells.

Next, the effect of MA, AK, and MIX on AICAR-induced myotube thickness reduction was investigated. After treatment of C2C12 myotubes with AICAR, the thickness of the myotubes was reduced as observed in the phase image (Fig. 2A). Meanwhile, MA, AK, and MIX significantly restored the AICAR-induced decrease in C2C12 myotube thickness (Fig. 2B). Additionally, we examined the effects of MA, AK, and MIX on gene expression changes following AICAR-induced atrophy. According to the results presented in Fig. 3, AICAR caused a slight but not significant decrease in the expression of *MyoD* and *myogenin*, but remarkably increased the expression of *atrogin-1* and *MuRF1*. However, MIX increased the expression of *MyoD* and *myogenin*, and decreased the expression of *atrogin-1* and *MuRF1*, which were increased by AICAR. These results confirmed that MIX could ameliorate AICAR-induced C2C12 myotube atrophy through regulating the expression of myogenesis and atrophy-related factors.

### MIX Restores the Expression of MyHC Reduced by AICAR

**Fig. 4:**
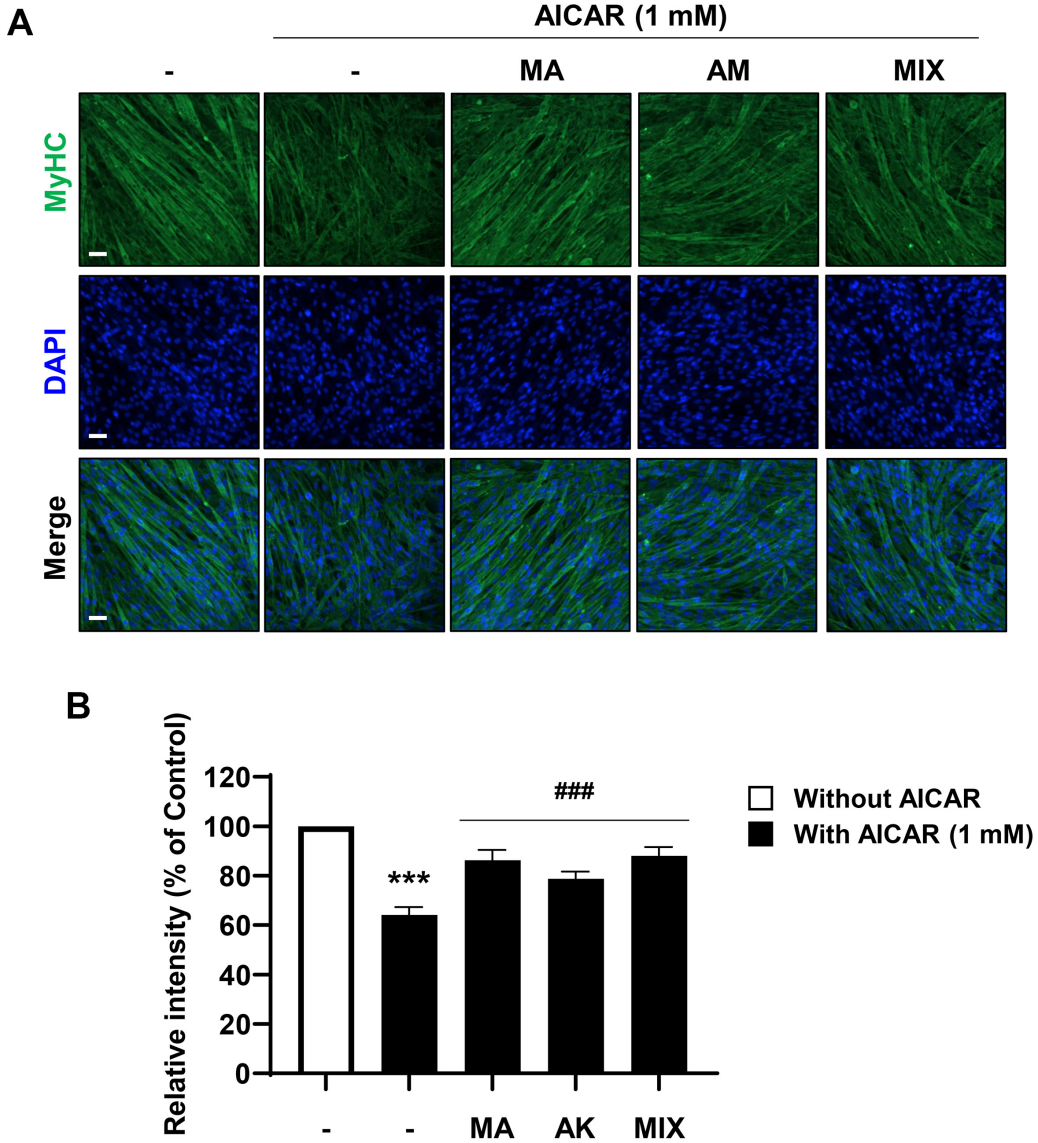
Effects of MA, AK, and MIX on MyHC suppressed by AICAR in C2C12 myotubes. (**A**) Immunofluorescence images of the expression of MyHC (Scale bar = 40 μm). (**B**) Fluorescence intensity data are expressed as the percentage of the control. ***p < 0.001 compared with the control. ^###^*p* < 0.001 compared with AICAR-treated cells.

MyHC is a major motor protein responsible for motility through muscle contraction. To determine the potential for functional recovery of muscle, the expression of MyHC was confirmed by immunofluorescence. It was observed that the fluorescence intensity of MyHC was decreased by AICAR. Moreover, as expected based on the previous results, treatment with MA, AK, and MIX increased the fluorescence intensity of MyHC, which was decreased by AICAR (Fig. 4). Therefore, MA, AK, and MIX are considered to be helpful in remediating skeletal muscle atrophy by improving the expression of motor proteins reduced by AICAR.

### MIX Improves Dexamethasone-Induced Muscle Atrophy in SD Rats

**Fig. 5:**
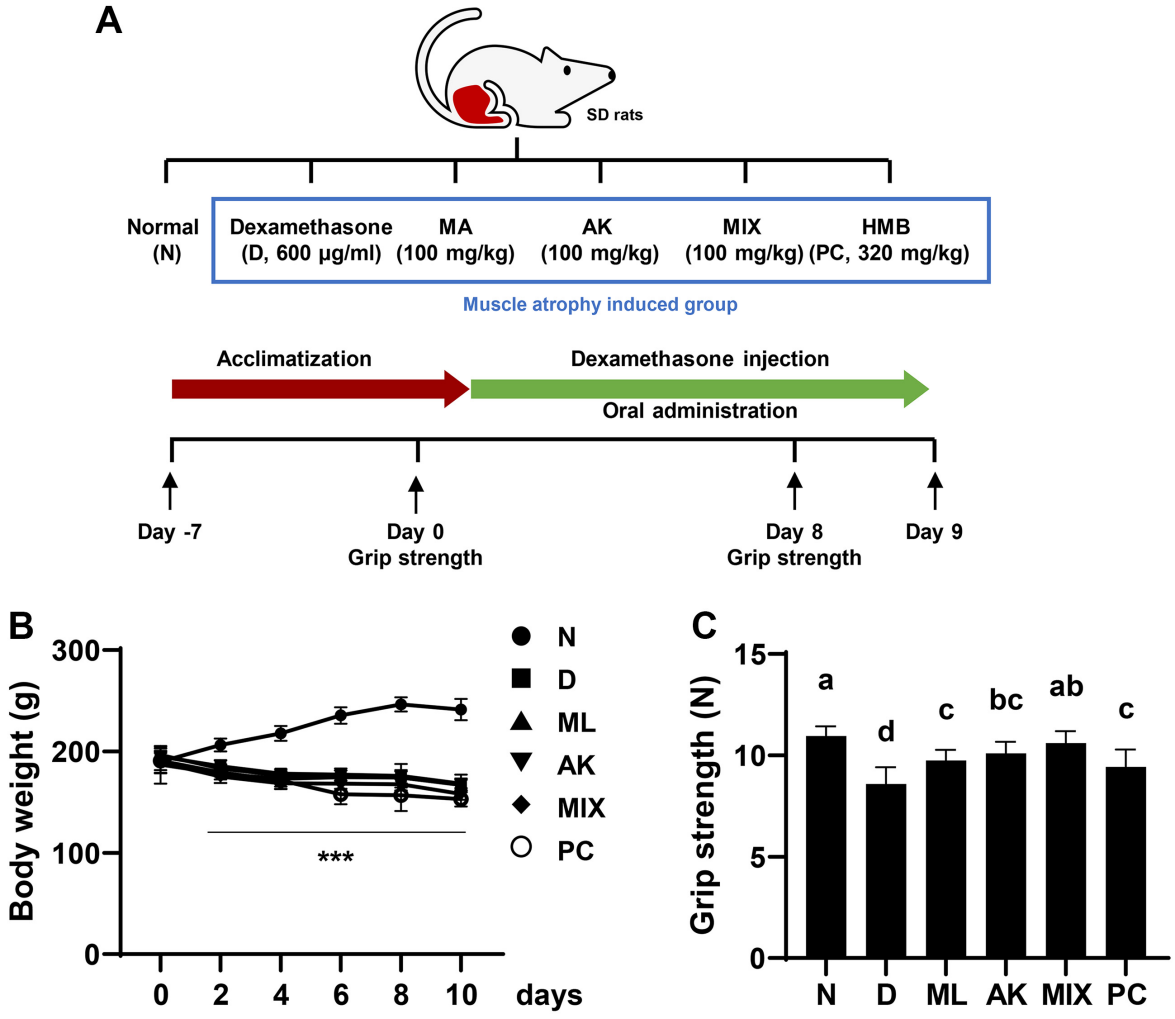
Effects of MA, AK, and MIX on dexamethasone-induced muscle atrophy in vivo. (**A**) The schematic diagram of animal experiment. (**B**) Body weight changes over 10 days. Data are expressed as means ± SD (*n* = 8). ***p < 0.001 compared with the normal group. (**C**) The grip strength of animals was measured. Different letters presented above the bars indicate significant differences (*p* < 0.05).

**Fig. 6:**
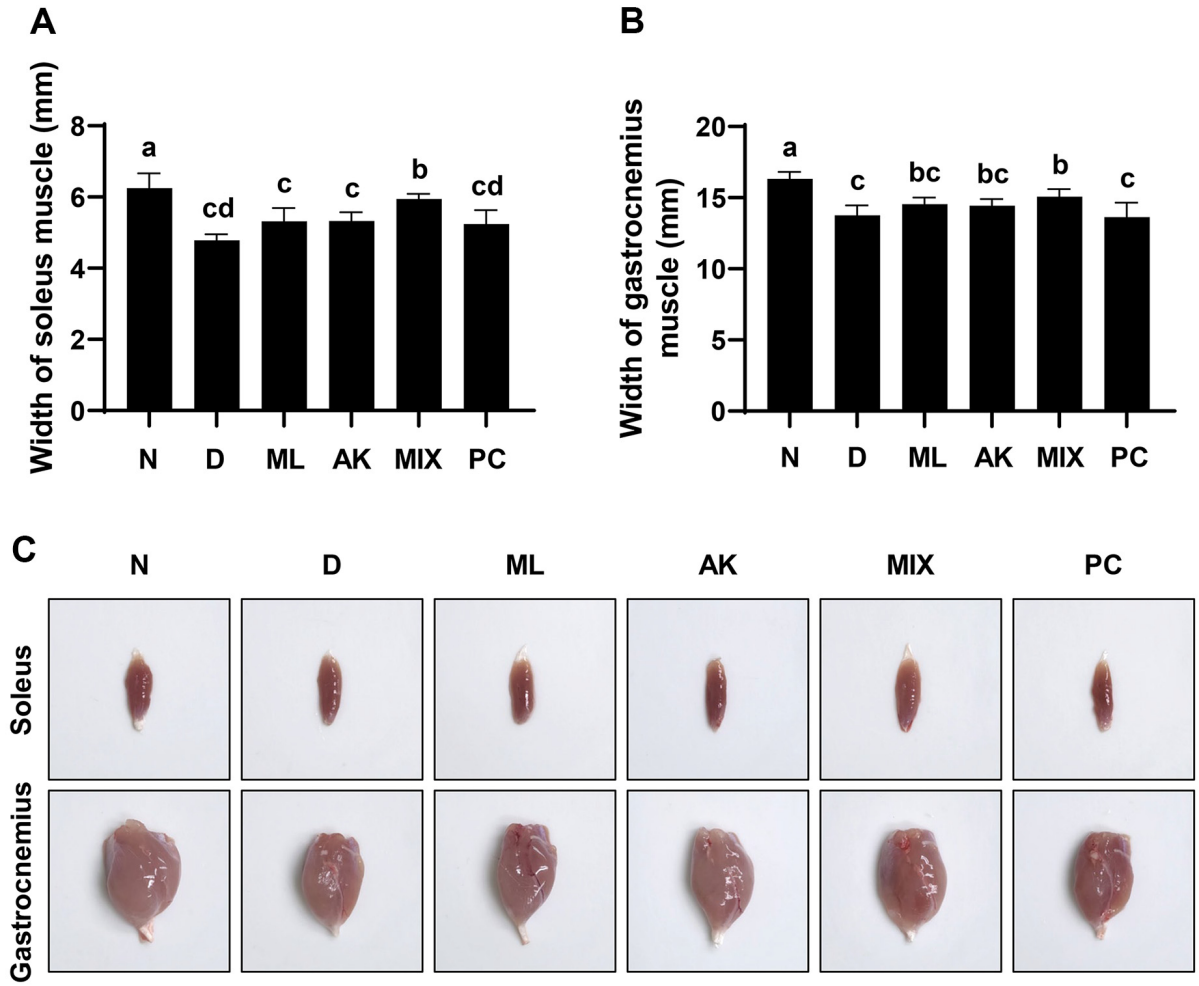
Effects of MA, AK, and MIX on the size of gastrocnemius and soleus muscles. Thickness of soleus (**A**) and gastrocnemius (**B**) muscles. Different letters presented above the bars indicate significant differences (*p* < 0.05). (**C**) Representative images of soleus and gastrocnemius muscles in each group.

Following up after the in vitro results, the efficacy of MIX on dexamethasone-induced muscle atrophy was investigated in vivo. The animals were administered dexamethasone (600 μg/kg/day) for 10 days, while MA, AK, and MIX were also administered for the same period. Body weight measurements of the animals during the experimental period were shown in Fig. 5B. In addition, a forelimb grip-strength test was performed to evaluate the functional improvement of the muscles by the MIX treatment. The results showed that the grip strength decreased by dexamethasone administration was significantly increased by MA, AK, and MIX administration (Fig. 5C). From the 2nd day of administration, all dexamethasone-administered groups showed a significant weight loss compared to the normal group. The thickness of the soleus and gastrocnemius muscles decreased in the dexamethasone-administered group, but the MIX-administered group showed significantly increased thickness, which was reduced by dexamethasone (Fig. 6). While the PC group showed no significant difference in the thickness of the gastrocnemius and soleus muscles, the grip strength that had been decreased by dexamethasone was partially restored. Therefore, these results indicated that MIX preserved dexamethasone-induced reduction in muscle mass and function.

### MIX Ameliorates Dexamethasone-Induced Muscle Histological Changes

**Fig. 7:**
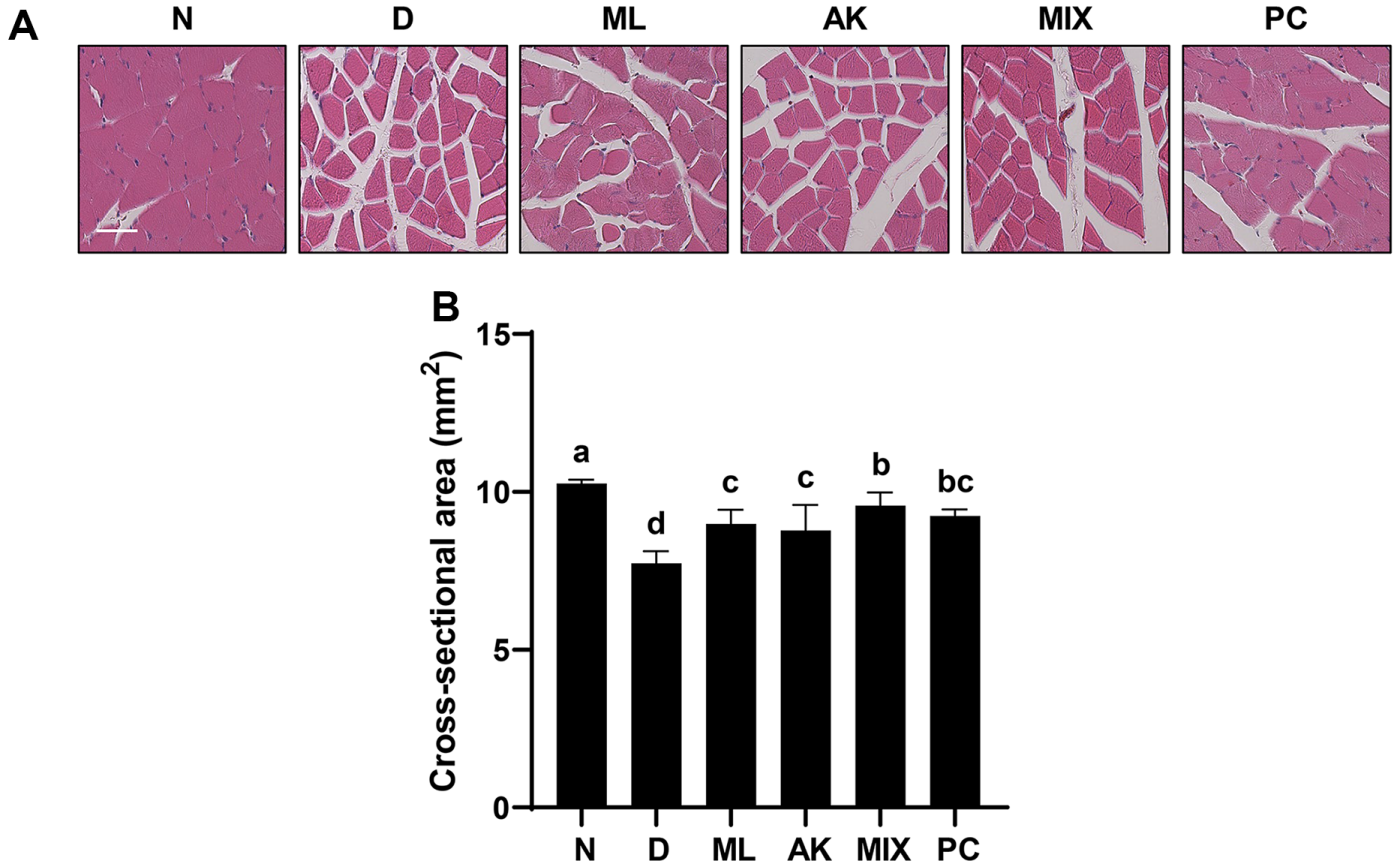
Effects of MA, AK, and MIX on histological changes of the gastrocnemius muscle induced by dexamethasone. H&E staining (**A**) and quantitative analysis of the cross-sectional area (**B**) of the gastrocnemius muscle (scale bar = 40 μm). Different letters presented above the bars indicate significant differences (*p* < 0.05).

To further examine the protective effect of MIX against dexamethasone-induced skeletal muscle atrophy, H&E staining of the gastrocnemius muscle tissue was performed. Compared to the normal group, the dexamethasone-treated group showed atrophy, large inter-fiber areas, loss of muscle fiber arrangement, and a decrease in the number of muscle fibers (Fig. 7A). However, these histological changes were reversed after MIX administration.

In addition, these histological changes were partially restored by the administration of MA and AK, and clear recovery was observed after the administration of MIX. Furthermore, the significant decrease in CSA in the dexamethasone group was reversed by an increase in CSA in the MIX group, supporting the increased thickness of the gastrocnemius muscle (Fig. 7B).

### MIX Regulates Myogenic Factors and Atrogenes in Rats with Dexamethasone-Induced Muscle Atrophy

**Fig. 8:**
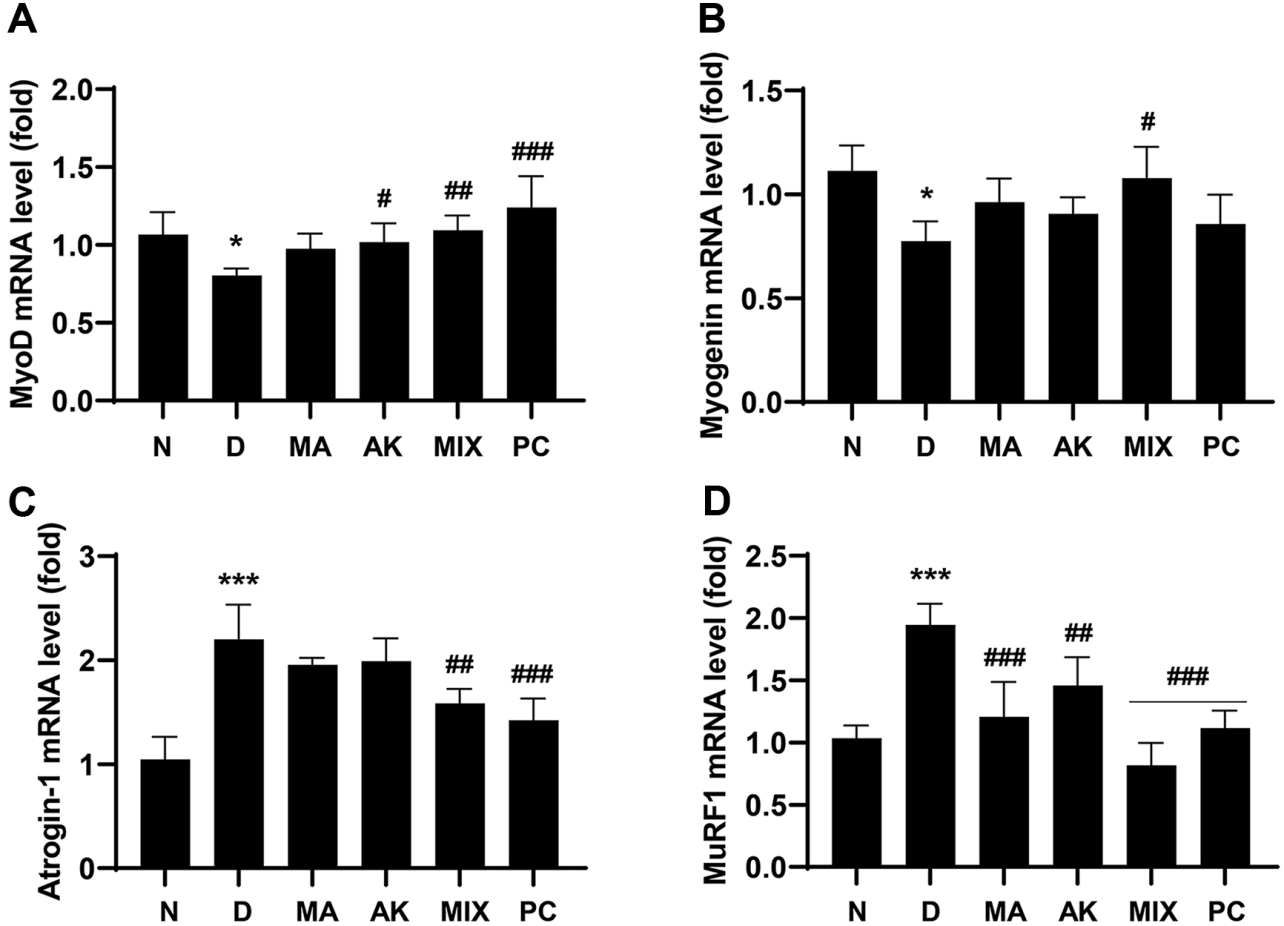
Effects of MA, AK, and MIX on the mRNA expressions levels of myogenic factors and atrophy-related factors in dexamethasone-induced muscle atrophy. Relative mRNA expressions levels of MyoD (**A**) myogenin (**B**) atrogin-1 (**C**) and MuRF1 (**D**) in gastrocnemius muscle were assessed by qRT-PCR. Data are expressed as means ± SD. *p < 0.05 and ***p < 0.001 compared with the normal group. ^#^*p* < 0.05, ^##^*p* < 0.01, and ^###^*p* < 0.001 compared with the dexamethasoneadministered group.

**Fig. 9:**
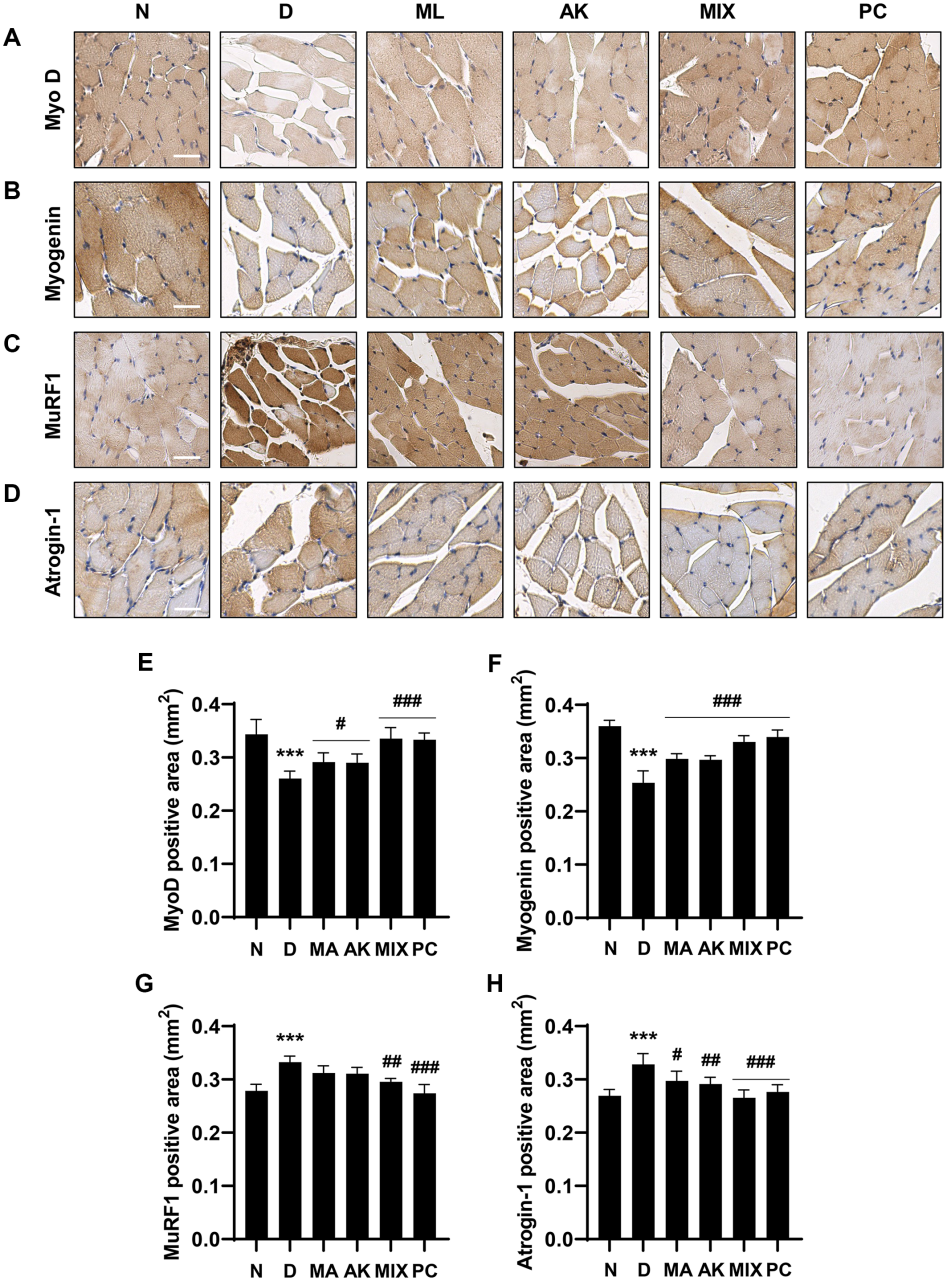
Effects of MA, AK, and MIX on the expression levels of myogenesis-related factors and atrophyrelated factors in dexamethasone-induced muscle atrophy. The expression levels of MyoD (**A**) myogenin (**B**) MuRF1 (**C**) and atrogin-1 (**D**) in gastrocnemius muscle were determined by immunohistochemistry (scale bar = 30 μm). Quantifications of immunohistochemistry staining of MyoD (**E**), myogenin (**F**), MuRF1 (**G**), and atrogin-1 (**H**). *p < 0.05 and ***p < 0.001 compared with the normal group. ^#^*p* < 0.05, ^##^*p* < 0.01, and ^###^*p* < 0.001 compared with dexamethasone-administered group.

The effect of MIX on dexamethasone-induced gene expression was investigated in gastrocnemius tissues. As shown in Fig. 8A and 8B, the mRNA expression levels of MyoD and myogenin, which were decreased by dexamethasone administration, were significantly upregulated by MIX administration. Moreover, the mRNA expression levels of *atrogin-1* and *MuRF1*, which are muscle-specific E3 ligases, were markedly increased by the administration of dexamethasone, whereas they were significantly decreased by the administration of MA, AK, and MIX (Fig. 8C and 8D). Furthermore, changes in protein expression level paralleled the changes in mRNA expression levels. In other words, the expression levels of *MyoD* and *myogenin*, which are myogenic factors, were reduced by dexamethasone, but restored by MIX. The expression levels of *atrogin-1* and *MuRF1*, which were downregulated by dexamethasone, were increased by MIX (Fig. 9).

### MIX Alleviates Dexamethasone-Induced Muscle Atrophy by Activating the Protein Synthesis Pathway

**Fig. 10:**
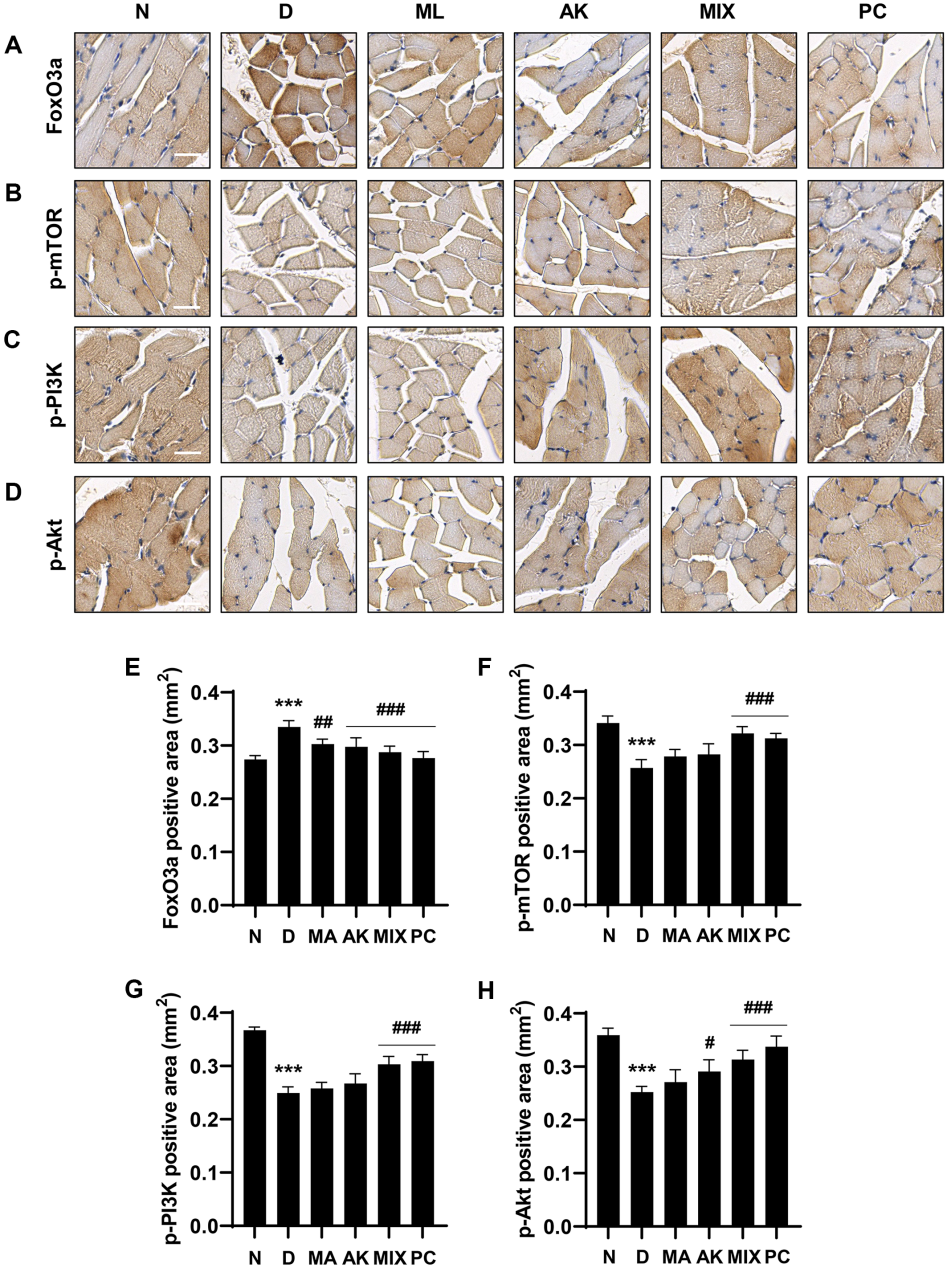
Effects of MA, AK, and MIX on the PI3K/Akt/mTOR signaling pathway and FoxO3a in dexamethasoneinduced muscle atrophy. The levels of FoxO3a (**A**), p-mTOR (**B**), p-PI3K (**C**), and p-Akt (**D**) were evaluated by IHC (Scale bar = 30 μm). Quantifications of immunohistochemistry staining of FoxO3a (**E**), p-mTOR (**F**), p-PI3K (**G**), and p-Akt (**H**). *p < 0.05 and ***p < 0.001 compared with the normal group. ^#^*p* < 0.05, ^##^*p* < 0.01, and ^###^*p* < 0.001 compared with the dexamethasone-administered group. N, normal group; D, dexamethasone (600 μg/kg)-treated group; ML, dexamethasone and ML (100 mg/kg)-treated group; AK, dexamethasone and AK (100 mg/kg)-treated group; MIX, dexamethasone and MIX (100 mg/kg)-treated group. PC, dexamethasone and 3-hydroxy-3-methylbutyric acid (320 mg/kg)-treated group.

Consequently, we measured changes in the expression levels of factors related to protein synthesis and degradation to determine their role in the effect of MIX on dexamethasone-induced muscle atrophy using immunohistochemistry. As shown in Fig. 10A, the expression level of FoxO3a was increased in the dexamethasone-treated group compared to the normal group, but was markedly reduced in the MIX-administered group. Moreover, dexamethasone decreased the levels of p-PI3K, p-Akt, and p-mTOR, and this effect was reversed by MIX (Fig. 10B-10D). These results suggest that MIX protects against muscle atrophy through the regulation of protein anabolic and catabolic processes.

## Discussion

Skeletal muscle atrophy is caused by various factors, such as disease and aging, but there is no clinically available treatment to date. Therefore, identifying agents that contribute to increased muscle mass and improved muscle function by balancing protein degradation/synthesis, as well as understanding the underlying mechanisms, are considered essential for treating muscle atrophy. In the present study, the efficacy of MIX (a mixture of MA and AK) in regulating protein catabolism- and anabolism-related factors was verified in vitro and in vivo.

AMPK plays an important role in maintaining cellular energy homeostasis; however, excessive AMPK activation leads to muscle atrophy due to decreased muscle protein synthesis through inhibition of the mTOR signaling pathway [11, 12, 14]. Our results showed that differentiated C2C12 myotubes were atrophied following treatment with AICAR, an AMPK inducer (Fig. 2). Myogenesis regulatory factors such as *MyoD*, *myogenin*, Mrf4, and Myf5 play major roles in the differentiation process in which C2C12 myoblasts undergo fusion to form myotubes [24, 37]. Among these, *MyoD*, along with Myf5, is a marker of the early stages of differentiation that induces the expression of its target gene, *myogenin* [38]. Our results showed that *myogenin* expression levels were increased by MIX treatment alone in C2C12 myotubes (Fig. 1B). In addition, the expression levels of *MyoD* and *myogenin*, which were decreased by AICAR treatment, were increased by MIX (Fig. 3A and 3B). Increased degradation of muscle proteins via the ubiquitin-proteasome system is a prominent mechanism that leads to muscle atrophy. In particular, muscle-specific ubiquitin ligases, *atrogin-1* and *MuRF1*, have been reported to be upregulated by AMPK activation [39]. Consistent with these results, in the present study, the expression levels of *atrogin-1* and *MuRF1* were significantly increased by AICAR treatment (Fig. 3C and 3D). Interestingly, MIX markedly suppressed the increased expression levels of agrogin-1 and *MuRF1* induced by AICAR treatment. Furthermore, a reduction in the levels of the myosin heavy chain, a major structural protein of myotubes, is a representative feature of muscle atrophy [40]. The results of this study showed that the level of MyHC expression, which was decreased by AICAR treatment, was increased by MIX treatment (Fig. 4). Therefore, MIX exerted a positive effect on the AICAR-induced atrophy of C2C12 myotubes by increasing the expression levels of factors involved in myogenesis and inhibiting markers of muscle atrophy that induce muscle protein degradation. Thus, MIX exerts a positive effect on AICAR-induced C2C12 myotube atrophy by regulating the expression of myogenesis- and muscle atrophy-related factors.

Although dexamethasone, a glucocorticoid, has a wide range of clinical uses including as an anti-inflammatory agent, it is considered to induce muscle atrophy by reducing the mass of skeletal muscle, one of the main target organs [17, 19]. Many studies have reported that dexamethasone induces thinning of muscle fibers through decreased muscle protein synthesis and increased degradation, resulting in decreased muscle strength due to a decrease in muscle mass [41-43]. MA attenuates insulin resistance in skeletal muscle by activating the insulin receptor substrate-1/PI3K/Akt signaling pathway [44], and flavonoids derived from MA have been associated with improvements in type 2 diabetes through enhanced mitochondrial function in the muscle [45]. According to previous studies, AK and compounds from AK inhibit muscle wasting by promoting myogenesis [46, 47]. In the present study, the effects of MIX, a mixture of MA and AK, on dexamethasone-induced muscle atrophy were verified in vivo. As reported in other studies, significant weight loss was observed after dexamethasone administration (Fig. 5B). While MIX administration did not change body weight, it significantly increased the thicknesses of the soleus and gastrocnemius muscles, which were reduced by dexamethasone (Fig. 6). Our results also showed that, as the thicknesses of the soleus and gastrocnemius muscles increased, the grip strength measurement results, which examined the functional aspects of the muscles, were improved by MIX administration (Fig. 5C). Additionally, dexamethasone administration was accompanied by histological changes such as atrophy of the muscle fibers and a corresponding increase in the inter-fiber gap and misalignment of the muscle fibers (Fig. 7). Consistent with the in vitro results, MIX increased the expression levels of myogenic factors, including *MyoD* and *myogenin*, and decreased the expression levels of E3 ubiquitin ligase-related factors, including *atrogin-1* and *MuRF1*, in an in vivo model of dexamethasone-induced muscle atrophy (Figs. 8 and 9). PI3K/Akt is considered to be a regulator required for the process of muscle formation and the survival of muscle cells [48]. In particular, the inhibition of PI3K/Akt signaling results in the dephosphorylation of FoxO3a, resulting in its translocation to the nucleus, where it promotes the expression of the E3 ubiquitin ligases, *atrogin-1* and *MuRF1* [20, 21, 49]. However, evidence suggests that PI3K/Akt activation is involved in myogenesis through increased activation of mTOR, a protein anabolic and catabolic regulator [14, 21, 50]. Our results showed that dexamethasone increased the levels of the dephosphorylated form of FoxO3a and decreased the levels of the phosphorylated form of mTOR (Fig. 10A and 10B). Moreover, the decreased levels of FoxO3a increased the levels of p-mTOR, as the activation of PI3K/Akt was increased by the administration of MIX (Fig. 10C and 10D).

Taken together, the present study demonstrated that MIX is effective against muscle atrophy through increased myogenesis factor expression levels and decreased expression levels of E3 ubiquitin ligase via modulation of the PI3K/Akt signaling pathway. Our results provide a rationale for the amelioration of dexamethasone-induced muscle atrophy through the regulation of the balance between protein synthesis and degradation. These findings suggest that MIX is a potential natural regulator that can be utilized for the treatment and prevention of muscle atrophy.

## Figures and Tables

**Table 1:** The list of primers used for qRT-PCR analysis.

Gene		5'→3' Primer sequence
*Myogenin*	Forward	CTA AAG TGG AGA TCC TGC GCA GC
	Reverse	GCA ACA GAC ATA TCC TCC ACC GTG
*MyoD*	Forward	ACT TTC TGG AGC CCT CCT GGC A
	Reverse	TTT GTT GCA CTA CAC AGC ATG
*MuRF1*	Forward	GTG TGA GGT GCC TAC TTG CTC
	Reverse	GCT CAG TCT TCT GTC CTT GGA
*iNOS*	Forward	CGT GTT TAC CAT GAG GCT GA
	Reverse	GCT TCA GGT TCC TGA TCC AA
*COX-2*	Forward	CAA AGG CCT CCA TTG ACC AG
	Reverse	TGG ACG AGG TTT TTC CAC CA
*Atrogin-1*	Forward	CAG CTT CGT GAG CGA CCT C
	Reverse	GGC AGT CGA GAA GTC CAG TC
*GAPDH*	Forward	GAA GAG TGG GAG TTG CTG TT
	Reverse	GGA GAA ACC TGC CAA GTA TG
