# Corrigendum to: A Mixture of Morus alba and *Angelica keiskei* Leaf Extracts Improves Muscle Atrophy by Activating the PI3K/Akt/mTOR Signaling Pathway and Inhibiting FoxO3a *In Vitro* and *In Vivo*

**DOI:** 10.4014/jmb.2026.3601.C01

**Published:** 2026-03-23

**Authors:** Hyun Hwangbo, Min Yeong Kim, Seon Yeong Ji, Da Hye Kim, Beom Su Park, Seong Un Jeong, Jae Hyun Yoon, Tae Hee Kim, Gi-Young Kim, Yung Hyun Choi

**Affiliations:** 1 Anti-Aging Research Center, Dong-eui University, Busan 47340, Republic of Korea; 2 Hamsoa Pharmaceutical Co., Ltd., Iksan 54524, Republic of Korea; 3 Department of Marine Life Science, Jeju National University, Jeju 63243, Republic of Korea; 4 Department of Biochemistry, Dong-eui University College of Korean Medicine, Busan 47227, Republic of Korea

The authors regret that the scientific name of *Morus alba* was incorrectly used in the title of the published article. The correct scientific name should be *Morus bombycis* Koidz. Therefore, the corrected title is presented below.

Furthermore, throughout the main text of the article, the plant described as *Morus alba* should be understood as *Morus bombycis* Koidz. The authors sincerely apologize for this oversight and any confusion it may have caused.

**Corrected Title:**

***A Mixture of Morus bombycis* Koidz. and *Angelica keiskei* Leaf Extracts Improves Muscle Atrophy by Activating the PI3K/Akt/mTOR Signaling Pathway and Inhibiting FoxO3a *In Vitro* and *In Vivo***

The authors would like to apologies for any inconvenience caused. (http://jmb.or.kr)

